# Process evaluation of five tailored programs to improve the implementation of evidence-based recommendations for chronic conditions in primary care

**DOI:** 10.1186/s13012-016-0473-8

**Published:** 2016-09-13

**Authors:** C. Jäger, J. Steinhäuser, T. Freund, R. Baker, S. Agarwal, M. Godycki-Cwirko, A. Kowalczyk, E. Aakhus, I. Granlund, J. van Lieshout, J. Szecsenyi, M. Wensing

**Affiliations:** 1 Department of General Practice and Health Services Research, University Hospital Heidelberg, Im Neuenheimer Feld 130.3, Turm West, 4. OG, 69120 Heidelberg, Germany; 2 University Hospital Schleswig-Holstein, Campus Lübeck, Institute of Family Practice, Ratzburger Allee 160, Haus 50, 23538 Lübeck, Germany; 3 Department of Health Sciences, University of Leicester, 22-28 Princess Road West, Leicester, LE16TP UK; 4 Medical Centre, Scientific Institute for Quality of Healthcare, Radboud University, PO Box 9101, 114 IQ Healthcare, 6500 HB Nijmegen, The Netherlands; 5 Centre for Family and Community Medicine, Medical University of Lodz, Kopcinskiego 20, 90-153 Lodz, Poland; 6 Research Center for Old Age Psychiatry in Innlandet Hospital Trust, N-2312 Ottestad, Norway; 7 Norwegian Knowledge Centre for the Health Services, Postboks 7004, St. Olavs plass, 0130 Oslo, Norway

**Keywords:** Tailoring, Process evaluation, Primary care, Implementation, Evidence-based, Guideline, Recommendations, Determinants of practice, Strategies

## Abstract

**Background:**

Although there is evidence that tailored implementation strategies can be effective, there is little evidence on which methods of tailoring improve the effect. We designed and evaluated five tailored programs (TPs) each consisting of various strategies. The aim of this study was to examine (a) how determinants of practice prioritized in the design phase of the TPs were perceived by health care professionals who had been exposed to the TPs and whether they suggested other important determinants of practice and (b) how professionals used the offered strategies and whether they suggested other strategies that might have been more effective.

**Methods:**

We conducted a mixed-method process evaluation linked to five cluster-randomized trials carried out in five European countries to implement recommendations for five chronic conditions in primary care settings. The five TPs used a total of 28 strategies which aimed to address 38 determinants of practice. Interviews of professionals in the intervention groups and a survey of professionals in the intervention and control groups were performed. Data collection was conducted by each research team in the respective national language. The interview data were first analyzed inductively by each research team, and subsequently, a meta-synthesis was conducted. The survey was analyzed descriptively.

**Results:**

We conducted 71 interviews; 125 professionals completed the survey. The survey showed that 76 % (*n* = 29) of targeted determinants of practice were perceived as relevant and 95 % (*n* = 36) as being modified by the implementation interventions by 66 to 100 % of professionals. On average, 47 % of professionals reported using the strategies and 51 % considered them helpful, albeit with substantial variance between countries and strategies. In the interviews, 89 determinants of practice were identified, of which 70 % (*n* = 62) had been identified and 45 % (*n* = 40) had been prioritized in the design phase. The interviewees suggested 65 additional strategies, of which 54 % (*n* = 35) had been identified and 20 % (*n* = 13) had been prioritized, but not selected in the final programs.

**Conclusions:**

This study largely confirmed the perceived relevance of the targeted determinants of practice. This contrasts with the fact that no impact of the trials on the implementation of the recommendations could be observed. The findings suggest that better methods for prioritization of determinants and strategies are needed.

**Trial registration:**

Each of the five trials was registered separately in recognized trial registries. Details are given in the respective trial outcome papers.

**Electronic supplementary material:**

The online version of this article (doi:10.1186/s13012-016-0473-8) contains supplementary material, which is available to authorized users.

## Background

It has been suggested that the transfer of evidence-based knowledge into practice would improve if the barriers to its adoption were overcome or facilitating factors used appropriately. “Tailoring” is a systematic approach to improve the design and effectiveness of interventions by selecting strategies explicitly to address specific, previously identified determinants of practice. Determinants of practice are factors that could influence the effectiveness of an intervention to improve professional practice and have been previously referred to using alternative terms, including barriers, obstacles, enablers, and facilitators [1]. Implementation strategies have been defined as “methods or techniques used to enhance the adoption, implementation and sustainability of a clinical program or practice” [2]. A range of frameworks have been suggested to classify determinants [3, 4] and implementation strategies [4–9], but none of them has been widely accepted, and although there is evidence that tailored interventions may have positive effects [1], it is unclear which methods most effectively identify and prioritize determinants and strategies [1, 10]. More evidence about the value of different methods would help developers of implementation programs improve health care practice.

In the project “Tailored Implementation for Chronic Diseases (TICD),” five tailored programs to implement recommendations for five different chronic health problems (chronic obstructive pulmonary disease, obesity, depression in the elderly, multimorbidity, and cardiovascular diseases) have been developed and evaluated in cluster-randomized controlled trials in Poland (PL), United Kingdom (UK), Norway (NW), Germany (GE), and The Netherlands (NL) [11]. An overview of the conditions and recommendations targeted and the strategies used in TICD is provided as Additional files 1 and 2.

The process of intervention development, which has been described previously in detail [12–14], comprised the systematic identification, prioritization, and selection of determinants potentially affecting the adoption by health professionals of the selected clinical practice recommendations. Subsequently, strategies to address the determinants were identified, prioritized, and selected. A combination of methods (brainstorming, interviews, focus groups, and a survey) involving various stakeholders (patients, physicians, researchers) was used to identify determinants and strategies. In a consensus procedure, prioritization involved the assessment of each determinant according to its assumed relevance and modifiability and the assessment of each strategy on its assumed impact and feasibility. The assessment was first done independently by at least two researchers using a scale from one to five for each criterion. In a second step, discrepancies were discussed to agree which of the potentially relevant and modifiable determinants should be addressed by the program, or which of the potentially effective and feasible strategies should be used.

**Fig. 1:**
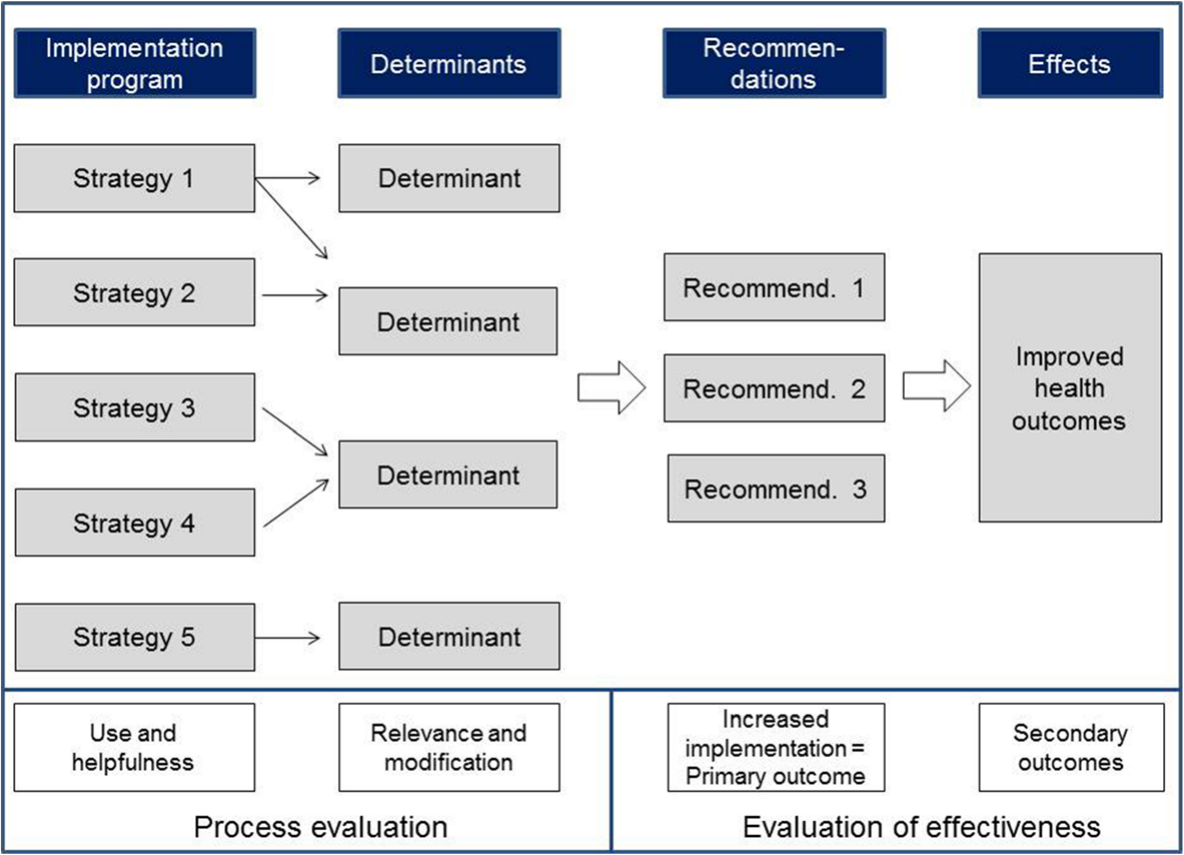
Logic model of the TICD trials. The figure describes the logic of the tailored programs developed within TICD: Implementation of evidence-based recommendations will improve if the applied strategies successfully modify previously identified determinants of practice. Since the recommendations are evidence based, i.e., their effectiveness has been substantiated, increased implementation will result in improved health outcomes. The content-specific logic models of each tailored program have been published in the respective study protocols [18–22]

Finally, a logic model specifying the assumed linkages between selected determinants and strategies was elaborated for each trial. The general structure of these models is depicted in Fig. 1.

Despite this detailed approach to developing the implementation interventions, none of the five studies achieved a positive effect on the primary outcome, which in all trials was the degree of the implementation of the recommendations measured by a set of indicators. The type and content of the indicators, however, was context-specific for each trial. A positive effect on some secondary outcomes could be observed [15–17] (Jaeger et al: Impact of a tailored program on the implementation of evidence-based recommendations for multimorbid patients with polypharmacy in primary care practices – results of a cluster-randomized controlled trial, under review in Implementation Science; Kowalczyk et al: Tailored implementation strategy to improve the management of patients with chronic obstructive pulmonary disease in primary care in Poland: a cluster randomized controlled trial, in preparation). While the primary and secondary outcomes of the programs differed across the trials [18–22], the process evaluation followed a common study protocol [23]. Its aim was to assess how the targeted health care professionals (HCP), i.e., the professionals who experienced the programs, viewed the choice of determinants to be addressed, and the strategies selected to address them. Furthermore, we intended to compare these views against the findings from the development phase of the intervention, during which interviews and surveys with stakeholders who had not experienced the programs had been conducted. In summary, the following research questions were addressed:

A. Research questions focusing on determinants:
  A1: Were the selected determinants perceived as relevant and modified by the targeted HCP?
  A2: Do the targeted HCP mention other important determinants which had not been identified or prioritized before?
B. Research questions focusing on strategies:
  B1: To what extent did the targeted HCP use and adapt the strategies offered by the tailored programs?
  B2: How helpful were the offered strategies from the perspective of the targeted HCP?
  B3: Should other strategies have been used from the perspective of the targeted HCP?

The questions were chosen to provide evidence on the usefulness of the methods used to identify and prioritize determinants and strategies in the context of tailoring.

## Methods

### Study design

This study was a process evaluation of five related cluster-randomized controlled trials of tailored implementation programs. We used a mixed-method approach consisting of semi-structured interviews and a survey to answer the research questions described above.

### Study population and recruitment

At the end of the intervention period, HCP of the intervention groups were invited to participate in an interview and/or the survey and HCP of the control groups were invited to participate in a survey. Recruitment for the intervention studies is described for each trial separately [15–17] (Jaeger et al: Impact of a tailored program on the implementation of evidence-based recommendations for multimorbid patients with polypharmacy in primary care practices – results of a cluster-randomized controlled trial, under review in Implementation Science; Kowalczyk et al: Tailored implementation strategy to improve the management of patients with chronic obstructive pulmonary disease in primary care in Poland: a cluster randomized controlled trial, in preparation). In Norway, only the interview study was conducted, as the survey was considered not feasible in the Norwegian trial.

### Data collection and processing

#### Interviews

Interviews were conducted separately in each trial in the respective national languages. All interviews followed a common semi-structured interview guide developed in English beforehand which contained the following questions:

1. It is recommended that … [introduce recommendation]. What do you think about this recommendation?
2. Where there any factors which made it difficult for you or helped you to adhere to the recommendation?
3. The implementation program consisted of various strategies. What strategies did you or other team members use and in what way? (If necessary, mention each strategy separately).
4. Did the implementation program help you to adhere to the recommendations?
  If yes, what strategies did you find helpful and why?
  If no, why not and what strategies would have been more helpful?

All interviews were audiotaped and transcribed. In Norway, the researchers involved in the design and delivery of the intervention conducted most of the interviews themselves; in the other countries (GE, NL, PL, UK) this was done by researchers not directly involved in the trial in order to avoid socially desirable responses.

#### Survey

The survey was conducted separately in each trial in the respective national languages but following a common framework elaborated in English (see Table 1). Item 1 of part 1 of the survey, which focused on determinants, was applied to the intervention and control groups. Item 2 of part 1 and part 2 of the survey focused on the use of the strategies by the HCP and was based on four aspects of adherence according to Carrol et al. [24]: content, duration, frequency, and coverage. It was applied to the intervention group only. The numbers of aspects formulated as items varied, because not all of them were applicable in the context of the various trials. Two additional items assessed adaptations to the strategies and their perceived helpfulness.

**Table 1:**
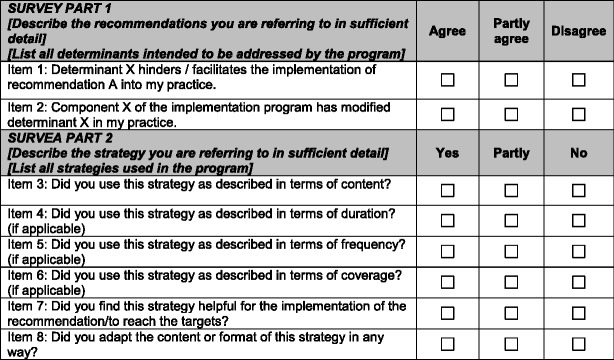
Framework of the survey

Item 1 was applied to the intervention and control groups; item 2 and part 2 were applied to the intervention groups only

### Data analysis

#### Interviews

Each research team analyzed the interviews individually following the principles of qualitative content analysis [25]. For this purpose, we used a common framework specifying the main categories for the analysis, which reflected the research questions of the process evaluation. Subcategories specific for the context of each trial were added inductively. Additionally, it was specified how frequently a theme was mentioned in the interviews using a coding from one to five (1 = strong issue in almost all interviews, 5 = single statement), whether the determinant or strategy respectively was identified and prioritized during the development phase of the intervention and whether the determinants were intended to be addressed by the implementation program. Subsequently, the emerging themes related to the determinants and the use of strategies were assigned to the TICD checklist, a comprehensive classification of determinants of practice developed in an earlier stage of the TICD project [3]. All teams used Atlas.ti software [26] for the coding.

After the country-specific analysis, each team provided the derived sets of categories with example quotations in English to the German team, who integrated them by merging identical codes and by harmonizing the assignments to the TICD checklist. This process was done in close collaboration with the researchers of the other teams to reduce the risk of bias. The framework for the interview analysis is depicted in Additional file 3.

Finally, the interview data from all countries were analyzed using a quantitative approach involving calculation of the percentage of the mentioned determinants that had been identified, prioritized (i.e., judged relevant and modifiable), and chosen to be addressed in the design phase of the intervention. Likewise, we calculated the percentage of strategies that had been identified, prioritized (i.e., judged effective and feasible), and selected.

#### Survey

The results of the survey are presented descriptively using means and percentages. The response categories “yes”/“partly” and “agree”/“partly agree” (see Table 1) were merged and interpreted as affirmation. The “majority of HCP” was defined as >66 % of professionals based on the consideration that this cutoff reflects the agreement or disagreement of a considerable number of HCP.

## Results

### Participants

In total, 71 interviews (9 in PL, 11 in UK, 12 in GE, 19 in NL, and 20 in NW) were conducted, and 125 primary HCP completed the survey. While in some countries, only general practitioners (GPs) were targeted by the survey or interviews; in other countries, practice nurses (PN) and health care assistants (HCA) were involved as well. In total, 211 HCP were invited for an interview, and 36 GPs, 26 PN, and 9 HCA participated, representing 33.6 % of the target group. The mean age of the interviewees was 44 years (ranging from 31 to 53 years across countries), and 70 % (*n* = 50) were female. The interviews lasted on average 34 (12–77) min. Socio-demographic characteristics of the survey respondents are depicted in Table 2.

**Table 2:** Type and number of HCP completing the survey on determinants

Trial	HCP completing the trial	HCP completing the survey	Response rate (%)	Profession % (*n*)	Mean age (years)	Female sex % (*n*)
	Total (IG/CG)	Total (IG/CG)^a^				
GE	21 (10/11)	21 (10/11)	100	GPs: 100 (21)	54.9 (44–68)	19.0 (4)
NL	33 (19/14)	30 (17/13)	90.9	PNs: 83 (25)	42.4 (22–61)	96.7 (29)
				HCA: 17 (5)		
PL	18 (9/9)	13 (7/6)	72.2	GPs: 100 (13)	47.6 (39–58)	46.2 (6)
					4 missings	4 missings
UK	146 (16/130)	61 (13/48)	41.8	GPs: 54 (33)	Not collected = 61 missings	Not collected = 61 missings
				PNs: 29 (18)		
				HCA: 15 (9)		
				Unknown: 2 (1)		
All	218 (54/164)	125 (47/78)	57.3	GPs: 54 (67)	48.3 (22–68)	31.2 (39)
				PNs: 34 (43)	65 missings	65 missings
				HCA: 10 (14)		
				Unknown: 1(1)		

^a^ *IG* intervention group, *CG* control group

### Results related to determinants (research questions A1 and A2)

Table 3 shows the results of part 1 of the survey focusing on determinants. The mean percentage of HCP answering the question, whether the targeted determinant was relevant for them, with “agree” or “partly agree” ranged from 60 to 93 % across trials. For item 2, asking whether the determinants were modified by the TP, the range was 83–98 %. From the 38 determinants presented in the survey and intended to be addressed by the five tailored interventions, 76 % (*n* = 29) were perceived as relevant and 95 % (*n* = 36) as modified by at least two thirds of the target group, although with substantial variation between countries (Table 4).

**Table 3:** Results of part 1 of the survey (focusing on determinants)

	Determinants intended to be modified by the program	Relevance*			Modification**
		Total	IG	CG	IG
		% (*n*)	% (*n*)	% (*n*)	% (*n*)
GE	1 Knowledge of HCP	71.4 (15)	80.0 (8)	63.6 (7)	100.0 (10)
	2 Routine	52.3 (11)	30.0 (3)	72.7 (8)	90.0 (9)
	3 Availability of medication lists	61.9 (13)	40.0 (4)	81.8 (9)	80.0 (8)
	4 Identification of the target group	23.8 (5)	40.0 (4)	9.0 (1)	90.0 (8)
	5 Feasibility of checklists	90.5 (19)	90.0 (9)	90.9 (10)	80.0 (8)
	6 Patients’ ability for self-management	90.5 (19)	90.0 (9)	90.9 (10)	80.0 (8)
	7 Language barrier	76.2 (16)	70.0 (7)	81.8 (9)	70.0 (7)
	8 Patients’ knowledge	57.1 (12)	60.0 (6)	54.5 (6)	90.0 (9)
	9 Standardization of medication lists	61.9 (13)	60.0 (6)	63.6 (7)	70.0 (7)
Mean of all items GE		65.1 (13.7)	62.2 (6.2)	67.6 (7.4)	83.3 (8.2)
NL	1 Apply motivational interviewing	93.3 (28)	100.0 (17)	84.6 (11)	100.0 (17)
	2 Giving good advice to patients	96.7 (29)	100.0 (17)	92.3 (12)	100.0 (17)
	3 More attention for the motivation of the patient	96.7 (29)	100.0 (17)	92.3 (12)	100.0 (17)
	4 PN gives lifestyle advice in an acceptable and feasible way	93.3 (28)	94.1 (16)	92.3 (12)	100.0 (17)
	5 PN meets patients’ information needs	93.3 (28)	94.1 (16)	92.3 (12)	94.1 (16)
	6 PN drafts feasible targets for patients	96.7 (29)	100.0 (17)	92.3 (12)	100.0 (17)
	7 E-health support for self-management	83.3 (25)	94.1 (16)	69.2 (9)	88.2 (15)
Mean of all items NL		93.3 (28)	97.5 (16.6)	87.9 (11.4)	97.5 (16.6)
PL	1 Availability of educational materials for recommendation 1	100.0 (13)	100.0 (7)	100.0 (6)	28.6 (2)
	2 Availability of training for GPs	69.2 (9)	57.1 (4)	83.3 (5)	42.9 (3)
	3 Labeling of medication records	100.0 (13)	100.0 (7)	100.0 (6)	100.0 (7)
	4 Accessibility of mMRC scale	100.0 (13)	100.0 (7)	100.0 (6)	100.0 (7)
	5 Accessibility of checklists for recommendation 2	92.3 (12)	85.7 (6)	100.0 (6)	100.0 (7)
	6 Availability of the recommendations	100.0 (13)	100.0 (7)	100.0 (6)	100.0 (7)
	7 Availability of training for personnel on dyspnea assessment	92.3 (12)	85.7 (6)	100.0 (6)	71.4 (5)
	8 Availability of educational materials for recommendation 3	100.0 (13)	100.0 (7)	100.0 (6)	100.0 (7)
	9 Availability of treatment plans	92.3 (12)	85.7 (6)	100.0 (6)	85.7 (6)
	10 Accessibility of checklist for recommendation 3	92.3 (12)	85.7 (6)	100.0 (6)	85.7 (6)
	11 Availability of peak flow meters	92.3 (12)	85.7 (6)	100.0 (6)	85.7 (6)
	12 Availability of demonstration inhalers	100.0 (13)	100.0 (7)	100.0 (6)	100.0 (7)
	13 Availability of educational materials on use of inhaler devices	100.0 (13)	100.0 (7)	100.0 (6)	100.0 (7)
	14 Training of GPs on inhaler devices	100.0 (13)	100.0 (7)	100.0 (6)	85.7 (6)
	15 Presence of additional staff (educator)	84.6 (11)	71.4 (5)	100.0 (6)	71.4 (5)
Mean of all items PL		94.4 (12.3)	90.5 (6.3)	98.9 (5.9)	83.3 (5.9)
UK	1 Skills of HCP to raise the issue of weight with patients	45.9 (28)	69.2 (9)	39.6 (19)	100.0 (13)
	2 Skills of HCP to measure waist circumference	50.8 (31)	53.8 (7)	50.0 (24)	92.3 (12)
	3 Skills of HCP to assess patients’ willingness to change	47.5 (29)	61.5 (8)	43.8 (21)	84.6 (11)
	4 Availability of resources to inform and motivate patients	67.2 (41)	84.6 (11)	62.5 (30)	100.0 (13)
	5 Availability of prescriptive weight loss information	83.6 (51)	92.3 (12)	81.3 (39)	100.0 (13)
	6 Work with HCP whom manage obese and overweight patients to improve their knowledge on diets	66.7 (32)	76.9 (10)	45.8 (22)	92.3 (12)
	7 Availability of information about referral pathways	59.0 (36)	76.9 (10)	54.2 (26)	92.3 (12)
Mean of all items UK		60.1 (35.4)	73.6 (9.6)	53.9 (25.9)	94.5 (12.3)

*IG* intervention group, *CG* control group, *GE* Germany, *NL* The Netherlands, *PL* Poland, *UK* United Kingdom

*Refers to item 1 of the framework depicted in Table 1

**Refers to item 2 of the framework depicted in Table 1. Numbers show the proportion of respondents who answered the respective questionnaire item with “agree” or “partly agree”

**Table 4:** Number of determinants perceived as relevant and modified by the tailored programs, per trial. Results of part 1 of the survey (focusing on determinants)

Number of determinants	GE	NL	PL	UK	All
Intended to be addressed	9	7	15	7	38
Perceived as relevant % (*n*)^a^	44 (4)	100 (7)	100 (15)	43 (3)	76 (29)
Perceived as modified % (*n*)^b^	100 (9)	100 (7)	87 (13)	100 (7)	95 (36)

*IG* intervention group, *CG* control group

^a^Number of determinants for which >66 % of respondents answered item 1 of the survey (see table 1) with “agree” or “partly agree”

^b^Number of determinants for which >66 % of respondents answered item 2 of the survey (see table 1) with “agree” or “partly agree”

In the interviews, 89 determinants for the recommendations were mentioned by the target group relating to 6 main and 25 of 57 subcategories of the TICD checklist (see Additional file 4). Categories with most items were “individual HCP factors” (21 determinants from 4 trials), “patient factors” (16 determinants from 4 trials), and “incentives and resources” (14 determinants from 4 trials). Of the 89 determinants, 70 % (*n* = 62) had been identified and 45 % (*n* = 40) had been prioritized during the development phase of the intervention. Thirty-seven percent (*n* = 33) of these determinants were intended to be addressed by the implementation programs, although there was wide variation between countries (Table 5).

**Table 5:** Comparison of determinants and strategies identified by interviews before and after the delivery of the program

	GE	PL	NW	UK	NL	All
	% (*n*)	% (*n*)	% (*n*)	% (*n*)	% (*n*)	% (*n*)
Number of determinants identified after program delivery	32	8	24	10	15	89
Thereof identified before	75 (24)	75 (6)	67 (16)	100 (10)	40 (6)	70 (62)
Thereof prioritized (i.e., judged to be relevant and potentially modifiable)	69 (22)	38 (3)	29 (7)	80 (8)	0	45 (40)
Thereof intended to be addressed by the program	50 (16)	38 (3)	25 (6)	80 (8)	0	37 (33)
Number of alternative strategies identified after program delivery	15	11	5	22	12	65
Thereof identified before % (*n*)	67 (10)	18 (2)	80 (4)	46 (10)	75 (9)	54 (35)
Thereof prioritized before (i.e., assessed as potentially effective and feasible, but not used in the final program)	20 (3)	18 (2)	0	18 (4)	33 (4)	20 (13)
Number and type of determinants not selected						
Patient factors	4	1	3	–	1	9
Capacity for organizational change	1	–	2	–	1	4
Incentives and resources	4	2	2	2	4	13
Professional interactions	1	1	1	–	3	6
Social, political, and legal factors	–	–	–	–	2	2
Individual health care professional factors	6	1	8	–	1	16
Not assigned to checklist	–	–	2	–	3	5
Number and type of strategies not prioritized						
Intervention development and delivery	–	6	–	3	–	9
Change service sites	3	–	1	3	1	8
Provision of materials	1	–	1	3	2	7
Share local knowledge	2	1	–	2	1	6
Change record systems	4	–	–	1	–	5
Training	1	–	1	3	–	5
Ongoing support	1	1	–	1	1	4
Adaptions on patient level	–	–	2	–	–	2
Revise professional roles	–	–	–	1	1	2
Funding	–	–	–	1	1	2
Provision of evidence	–	–	–	–	1	1
External feedback	–	1	–	–	–	1

This table shows the quantitative analysis of the qualitative data presented in Additional file 4

### Results related to the use of strategies (research questions B1 and B2)

Table 6 shows the results of part 2 of the survey and the percentages of respondents per country who positively answered the questions on whether they had used the respective strategy. The mean of all items varied between countries and ranged from 31 % (NL) to 57 % (GE). The proportion of respondents who reported adapting one or several strategies in practice also varied between strategies and countries but was overall low. One item in the questionnaire asked whether the respective strategies were perceived as helpful by the target group. On average, 33 % (NL)–77 % (UK) of the respondents answered this item in the affirmative; for individual strategies, variation was between 0 and 94 %. The majority of respondents in all trials appreciated face-to-face training sessions.

**Table 6:** Results of part 2 of the survey (focusing on the use of strategies)

Country	Strategy^a^ (short term)	Use of the strategy in terms of^b^					Adaption^c^	Helpfulness^d^
		Content	Frequency	Duration	Coverage	Total		
GE (*n* = 10)	GE 1	100.0	n.a.	n.a.	n.a.	100.0	n.a.	60.0
	GE 2	100.0	n.a.	n.a.	n.a.	100.0	0.0	60.0
	GE 3	30.0	n.a.	n.a.	n.a.	30.0	10.0	80.0
	GE 4	70.0	n.a.	n.a.	n.a.	70.0	10.0	60.0
	GE 5	40.0	n.a.	n.a.	n.a.	40.0	0.0	90.0
	GE 6	50.0	n.a.	n.a.	n.a.	50.0	10.0	50.0
	GE 7	50.0	n.a.	n.a.	n.a.	50.0	0.0	40.0
	GE 8	20.0	n.a.	n.a.	n.a.	20.0	10.0	50.0
	GE 9	50.0	n.a.	n.a.	n.a.	50.0	0.0	40.0
	Mean of all items GE					56.7	5.0	58.9
UK (*n* = 13)	UK 1	100.0	n.a.	n.a.	n.a.	100.0	23.1	84.6
	UK 2	53.8	30.8	n.a.	30.8	38.4	0	53.8
	UK 3	84.6	69.2	n.a.	61.5	71.8	7.7	92.3
	UK 4	61.5	n.a.	n.a.	46.2	53.9	7.7	76.9
	UK 5	61.5	38.5	n.a.	61.5	53.8	7.7	84.6
	UK 6	69.2	15.4	0.0	38.5	41.0	7.7	76.9
	UK 7	30.8	n.a.	n.a.	n.a.	30.8	7.7	69.2
	Mean of all items UK					55.7	8.8	76.9
PL (*n* = 7)	PL1	71.4	n.a.	n.a.	n.a.	71.4	n.a.	n.a.
	PL2	n.a.	57.1	28.6	14.3	33.3	0	28.6
	PL3	n.a.	28.6	14.3	14.3	19.0	28.6	28.6
	PL4	n.a.	28.6	42.9	71.4	47.6	28.6	42.9
	Mean of all items PL					42.8	19.1	33.4
NL (*n* = 17)	NL1	100.0	n.a.	n.a.	n.a.	100.0	n.a.	94.1
	NL2	64.7	n.a.	n.a.	n.a.	64.7	n.a.	64.7
	NL3	17.6	n.a.	n.a.	n.a.	17.6	n.a.	17.6
	NL4	11.8	n.a.	n.a.	17.6	14.7	23.5	11.8
	NL5	5.9	n.a.	n.a.	n.a.	5.9	23.5	41.2
	NL6	5.9	n.a.	n.a.	n.a.	5.9	n.a.	0
	NL7	23.5	n.a.	n.a.	n.a.	23.5	47.1	23.5
	NL8	17.6	n.a.	n.a.	n.a.	17.6	35.3	11.8
	Mean of all items NL					31.2	32.3	33.1

^a^For explanation of the short terms, see Additional file 2

^b^Refers to item 3–6 of the framework depicted in Table 1

^c^Refers to item 8 of the framework depicted in Table 1

^d^Refers to item 7 of the framework depicted in Table 1. Numbers represent percentages of respondents who answered the respective questionnaire item in the affirmative

In the interviews, we identified 19 common themes related to reasons why the participants considered a strategy helpful or not, or used them or not (see Additional file 5). All themes could be assigned to five main and 14 subcategories of the TICD checklist. The majority of themes referred to the extent to which the strategy addressed individual health professional factors (such as knowledge, awareness, attitudes, motivation, and behavior) and the characteristics of the recommended strategy (such as accessibility, feasibility, effort, compatibility, and quality of underlying evidence). The extent to which the strategy influenced professional interactions, patient factors, and incentives and resources were other domains related to the use of the strategies.

### Results related to alternative strategies (research question B3)

Additional file 6 presents the other strategies suggested by the interview participants that had not been included in the respective implementation program, which they saw as likely to be helpful. As Table 5 shows, 65 alternative strategies were suggested. Of these, 54 % (*n* = 35) had been identified in the intervention development phase and 20 % (*n* = 13) had been prioritized. Suggestions which had not been prioritized most frequently related to the development and delivery of the intervention, to changes of service sites, the provision of materials, and the sharing of local knowledge.

## Discussion

The assumption underlying the concept of tailoring is that implementation is more likely to be successful if strategies are selected to address previously assessed determinants of practice and the needs and preferences of the target group. The results presented in this paper partly support this assumption.

This study largely confirmed that the targeted HCP, after experiencing the programs, perceived the determinants which we identified and selected by various methods in the design phase of the implementation program as relevant. The majority of strategies were perceived as having at least partly modified the determinants. This suggests that a mix of interviews and surveys, involving a range of stakeholders, can effectively identify important determinants of practice and potential strategies for addressing them.

However, we also found that about 30 % of the determinants mentioned by HCP who had experienced the interventions had not been identified and 55 % had not been prioritized during the design of the programs. About one quarter of the determinants intended to be addressed were perceived as not relevant by the majority of the HCP. The proportion of respondents who had used the tailored strategies and considered them helpful varied across strategies and trials but was often less than two thirds. There was little evidence of adaptation of the strategies during application by targeted HCP. Many additional strategies were suggested, some of which had been mentioned in the intervention development phase but had not been selected in the programs.

This suggests that the methods for the prioritization of determinants and strategies should be improved. Our prioritization process was based on the assumed relevance and modifiability of the determinants and the assumed impact and feasibility of the strategies, meaning that determinants and strategies which were perceived as not being modifiable or feasible, respectively, were not selected. Yet such apparently unmodifiable determinants or unfeasible strategies may be decisive for successful implementation. Furthermore, continued monitoring of the experiences of the HCP during program delivery and program adaptation to newly emerging determinants may be needed. The latter has to be balanced against the cost of such monitoring and the need to standardize interventions in the context of rigorous evaluation research.

The fact that the linkages of the logic models were confirmed by a substantial number of HCP contrasts with the limited effect on the primary outcomes of the trials. There are several possible explanations for this discrepancy: We chose pragmatically to design models with linear relationships between determinants, strategies, and effects. Although these models were useful to guide the development and evaluation of the five interventions, possibly more advanced models with more complex (e.g., nonlinear) relationships incorporating theories of behavior change, such as the theoretical domains framework [27], or learning theories as well as adaption of the program theory may be needed to specify valid cause-effect chains, as postulated by several authors [28–31]. In this context, the classification of implementation strategies is important. Implementation strategies have been defined as “methods or techniques used to enhance the adoption, implementation and sustainability of a clinical program or practice” [2]. A growing body of literature deals with the challenges of labeling and reporting implementation strategies [9, 32]. Several lists and taxonomies attempting to reflect the range of strategies have been published [4–9, 33], but none is widely accepted and none of them was sufficiently applicable to our programs. It was also striking that even researchers in the same project (TICD) specified strategies in different levels of detail (see Additional file 2), and so it is unclear whether the assumed mechanism leading to the desired effect was really understood and well communicated to the participants. This reflects the need for better frameworks for the specification of implementation strategies, which comprise different levels of abstraction, for instance by distinguishing the mode of delivery (e.g., workshop), the technique (e.g., role plays), and the aim of the intervention (e.g., to raise awareness, to convey knowledge) [34].

Some studies suggest that other variables, such as the length of follow up, the number of intervention contacts, the type of participant population, and demographics, may moderate the effect of tailored interventions, [30] and may be more decisive than the methods used for the identification of determinants. Yet studies evaluating tailored vs. non-tailored interventions are still few in number and more should be undertaken in the future [1]. Factors related to the research design, such as the chosen outcomes or the sample size, may have been other reasons why no impact on the primary outcomes was detected.

Some strengths and limitations of this study should be taken into account. The process evaluation of all trials followed a common, previously published study protocol [23]. We synthetized data from five trials focusing on five different health problems, all following the same process of tailoring. On one hand, this increases the generalizability of the results. On the other hand, the necessary standardization of the process evaluation including the translation of the results into English might have influenced the precision of the interpretations. The survey was conducted among a small number of HCP and only in four out of the five trials, since it was not considered feasible in Norway, meaning that the results may be susceptible to outliers. The high variance of the responses between and within countries makes it difficult to draw general conclusions. Since we involved different methods and target groups to identify determinants before and after the programs were delivered, comparability is limited. Also we did not rate the relevance and modifiability of the determinants and the feasibility of the strategies suggested after the delivery of the intervention taking self-reported views of the health care professional who had experienced the programs as “gold standard” against which to compare the determinants identified before the delivery of the intervention. However, the health professionals themselves may have had only a limited understanding of the determinants despite having experienced the program.

## Conclusions

The linkages between determinants of practice and strategies hypothesized by the logic models of the interventions have been confirmed by the majority of the target group, suggesting that a combination of methods and stakeholders effectively identifies relevant determinants and appropriate strategies. However, this did not result in a measurable improvement of the implementation of the recommendations, indicating that other factors such as the use of theory, monitoring of experiences, intervention fidelity, adaptions of the interventions to individual, and contextual factors or improved outcome measures are equally decisive for the effect of an intervention than the methods used for tailoring. Future research should focus on comparisons between tailored and non-tailored interventions. The process of tailoring should be improved by developing better methods to prioritize determinants of practice and strategies and comprehensive frameworks to standardize the reporting of strategies.

## Abbreviations

GE, Germany; GP, general practitioner; HCA, health care assistant; HCP, health care professional; NL, The Netherlands; NW, Norway; PL, Poland; PN, practice nurse; TICD, Tailored Implementation for Chronic Diseases; UK, United Kingdom

## Additional files

Additional file 1: Chronic conditions and recommendations targeted in the TICD project. (PDF 188 kb)

Additional file 2: Strategies used in the TICD trials. (PDF 213 kb)

Additional file 3: Framework for the interview analysis. (PDF 15 kb)

Additional file 4: Determinants identified by interviews after the delivery of the intervention. (PDF 175 kb)

Additional file 5: Results of the qualitative analysis (aspects related to the use of the strategies). (PDF 166 kb)

Additional file 6: Results of the qualitative analysis (other strategies not used in the implementation programs). (PDF 209 kb)
