# Supplementary material for: Process evaluation of five tailored programs to improve the implementation of evidence-based recommendations for chronic conditions in primary care
Source: Implement Sci. 2016 Sep 13;11:123. doi: 10.1186/s13012-016-0473-8 (PMC5022166; doi:10.1186/s13012-016-0473-8)
Supplement: Supplementary file 1 — Chronic conditions and recommendations targeted in the TICD project. (PDF 188 kb) [file 13012_2016_473_MOESM1_ESM.pdf]

Process evaluation of five tailored programs to improve the implementation of evidence-based recommendations for chronic conditions in primary care

C. Jäger, J. Steinhäuser, T. Freund, R. Baker, S. Agarwal, M. Godycki-Cwirko, A. Kowalczyk, E. Aakhus, I. Granlund, J. van Lieshout, J. Szecsenyi and M. Wensing

Additional file 1 - Chronic conditions and recommendations targeted in the TICD-project

Country	Targeted chronic condition	Recommendations chosen as implementation objectives
Germany (GE)	Multimorbidity (polypharmacy)	1. **Structured medication counseling incl. brown bag review:** should be offered to multimorbid patients with polypharmacy and additional risk factors at least once per year<br>2. **Medication lists:** should meet minimum standards concerning layout and template and patients with permanent medication should always have them with them.<br>3. **Medication reviews** should be conducted by GPs using instruments to reduce potentially inappropriate medication
The Netherlands (NL)	Cardiovascular Diseases (CVD)	1. **SBP < 140 mmHg** in patients at high risk for CVD<br>2. **SBP < 140 mmHg** in patients with established CVD<br>3. **LDL < 2,5 mmol/l** in patients at high risk for CVD<br>4. **LDL < 2,5 mmol/l** in patients with established CVD<br>5. **Promote life-style-changes** in patients with (high risk for) CVD<br>6. **Create a risk-profile** for patients with chronic kidney disease
Poland (PL)	COPD	**1.** **A brief counseling to quit smoking**: should be offered to all patients with COPD at least once a year.<br>**2.** **Assess prognosis of COPD using mMRC dyspnea scale**: All patients with COPD should have the degree of dyspnea assessed by the mMRC scale at least once a year and results recorded in their medical record.<br>**3.** **Inform patients about COPD following a checklist**: Physicians should discuss the specific components of the care process with all patients with COPD.<br>**4.** **Train patients in the correct use of inhaler devices**
United Kingdom (UK)	Obesity	1. **Use BMI or waist circumference**: to determine the degree of overweight or obesity<br>2. **Assessment**: of lifestyle, co-morbidities and willingness to change<br>3. **Management of obesity:** A multi-component intervention should be offered to encourage increased physical activity, improved eating behaviour, and healthy eating. Drugs may be used in certain groups. The intervention should involve long-term follow up by a trained professional and be tailored to the patient's preferences, initial fitness and lifestyle.<br>4. **Referral to specialists:** if the cause is uncertain, if conventional treatment has failed and surgery is being considered or if there are complex co-morbidities and specialist intervention are needed.
Norway (NW)	Depression in the elderly	1. **Social contact:** should be discussed with elderly patients with depression and actions to increase social contact recommended if needed<br>2. **Collaborative care plans:** should be developed by municipalities for patients with moderate and severe depression.<br>3. **Depression Care Manager:** PCP should offer regular contact with a Depression Care Manager<br>4. **Counseling:** PCP should offer advice regarding self-assisted programs on behavioural therapy, physical activity groups, sleeping habits, anxiety coping strategies and problem solving therapy<br>5. **Mild depression:** Avoid routine prescription of antidepressants<br>6. **Severe, recurrent or chronic depression or dysthymia:** Offer a combination of antidepressants and psychotherapy.

GP = general practitioner, SPB = systolic blood pressure, CVD = cardiovascular diseases, LDL = low density lipoproteine, COPD = chronic obstructive pulmonary disease, BMI = body mass index, PCP = primary care practices, mMRC = Modified British Medical Research Council
