# Supplementary material for: Process evaluation of five tailored programs to improve the implementation of evidence-based recommendations for chronic conditions in primary care
Source: Implement Sci. 2016 Sep 13;11:123. doi: 10.1186/s13012-016-0473-8 (PMC5022166; doi:10.1186/s13012-016-0473-8)
Supplement: Supplementary file 2 — Strategies used in the TICD trials. (PDF 213 kb) [file 13012_2016_473_MOESM2_ESM.pdf]

Process evaluation of five tailored programs to improve the implementation of evidence-based recommendations for chronic conditions in primary care

C. Jäger, J. Steinhäuser, T. Freund, R. Baker, S. Agarwal, M. Godycki-Cwirko, A. Kowalczyk, E. Aakhus, I. Granlund, J. van Lieshout, J. Szecsenyi and M. Wensing

Additional file 2 - strategies used in the TICD trials

**Groups of strategies**		**Original phrasing of the strategies**	**Short term / country**
Educational sessions	Educational group meetings	Workshop for GPs and HCA on medication management	GE1
		Face-to-face training for HCP on how to raise the issue of weight with patients	UK1
		Face-to-face training for HCP to improve their knowledge on the management of obese and overweight patients	UK6
		Face-to-face training for HCP on waist circumference measurement and interpretation	UK2
		Training of physicians in identifying the smoking status, in anti-smoking counseling	PL1
	Educational outreach visits	Refresher training in motivational interviewing for practice nurses	NL1
		Outreach visits to general practitioners	NW1
	Induce knowledge exchange between peers	Discussion of the quality improvement concepts in the quality circle	GE3
Educational materials	E-learning tools for HCP and patients	Online CME course on depression	NW2
		“Info-tool” on a tablet PC to inform patients about medication-related topics	GE4
		E-learning module on CV risk management for PN	NL2
		Twitter consultations for patients	NL6
	Increase the accessibility of web resources	Web resources to facilitate medication reviews provided on a tablet PC	GE3
		Creation of a website with various tools to diagnose, follow-up and council patients with depression	NW3
		Support PN to use e-health applications for patients without symptoms of depression	NL4
	Tools for patient counseling	Resources to demonstrate the benefits of a modest 5 – 10% weight loss	UK4
		Provision of demonstration inhalers	PL4
	Printed information material to be handed out to individuals	Weight-loss-you-can-see booklets	UK5
		“Brown bags” with imprints encouraging patients to bring their medication packages to the practice	GE6
		Templates, manuals and pamphlets to be distributed to patients, their relatives and healthcare professionals in the municipality	NW4
	Information campaign	Posters for the waiting room to raise awareness among patients	GE5
	Brief summaries	Provision of questions/scripts for HCP to use to assess patients motivation and readiness to lose weight	UK3
		Provision of summary of the NICE guidelines for diet and exercise to all HCP	/
		Insertion of a printed form containing the modified Medical Research Council Dyspnea Scale (mMRC) into patients’ medical records.	PL2
		Provision of a checklist for practitioners defining the elements which consultation of patients with COPD should contain	PL3
		Provision of a summary of the evidence underlying the recommendations	NW5
		Provision of a screening instrument for depression	NL3
		Provision of cards with treatment values	NL5
		Provision of a checklist for medication counseling	GE7
		Provision of a checklist for medication reviews	GE 8
		Provision of a template for minimum standards of medication lists	GE 9
Induce organizational change	Elaboration of individual implementation concepts (tailoring on individual level)	Arrange team meetings to elaborate a quality improvement concept for each practice	GE2
		Support for the development collaborative care plans per community, with an option to tailor interventions to the community	NW6
	Support for referral processes	Support for PN to refer patients with mild/severe symptoms of depression to physical activity groups or depression treatment respectively	NL7/NL8
		To work with health professionals from each practice to create a database of local programmes patients can be referred to	UK7

GP = general practitioner, HCA = health care assistant, HCP = health care professional, CME = continuous medical education, PN = practice nurses, NICE = National Institute of Clinical Excellence, mMRC = Modified British Medical Research Council, COPD = chronic obstructive pulmonary disease, CV= cardiovascular, GE = Germany, NL = The Netherlands, NW = Norway, UK = United Kingdom, PL = Poland
