# Supplementary material for: Process evaluation of five tailored programs to improve the implementation of evidence-based recommendations for chronic conditions in primary care
Source: Implement Sci. 2016 Sep 13;11:123. doi: 10.1186/s13012-016-0473-8 (PMC5022166; doi:10.1186/s13012-016-0473-8)
Supplement: Supplementary file 3 — Framework for the interview analysis. (PDF 15 kb) [file 13012_2016_473_MOESM3_ESM.pdf]

Process evaluation of five tailored programs to improve the implementationof evidence-based recommendations for chronic conditions in primary care

C. Jäger, J. Steinhäuser, T. Freund, R. Baker, S. Agarwal, M. Godycki-Cwirko, A. Kowalczyk, E. Aakhus, I. Granlund, J. van Lieshout, J. Szecsenyi and M. Wensing

Additional file 3: Framework for the interview analysis

Main category (deductive)	Sub-category (inductive)	Description	Example quotation	Frequency (1-5) *	Identified before? yes/no	Prioritised before? yes / no	TICD checklist category
Determinants for implementation							
Other suggested strategies not used in the implementation program							n.a.
Reasons why strategy was used as planned / considered helpful					n.a.	n.a.	
Reasons why strategies were not used as planned / not considered helpful					n.a.	n.a.	
Reasons why strategies were used differently than planned / adapted					n.a.	n.a.	

* 1 = strong issue in almost all interviews, 2 = strong issue in a few interviews, 3 = side issue in almost all interviews, 4 = side issue in a few interviews, 5 = single statement
