# Supplementary material for: Process evaluation of five tailored programs to improve the implementation of evidence-based recommendations for chronic conditions in primary care
Source: Implement Sci. 2016 Sep 13;11:123. doi: 10.1186/s13012-016-0473-8 (PMC5022166; doi:10.1186/s13012-016-0473-8)
Supplement: Supplementary file 4 — Determinants identified by interviews after the delivery of the intervention. (PDF 175 kb) [file 13012_2016_473_MOESM4_ESM.pdf]

Process evaluation of five tailored programs to improve the implementation of evidence-based recommendations for chronic conditions in primary care

C. Jäger, J. Steinhäuser, T. Freund, R. Baker, S. Agarwal, M. Godycki-Cwirko, A. Kowalczyk, E. Aakhus, I. Granlund, J. van Lieshout, J. Szecsenyi and M. Wensing

Additional file 4: Determinants identified by interviews after the delivery of the intervention

TICD checklist		Determinant original phrasing	T*	F*	IB*	PB*	IA*
Patient factors	Patient preferences	Patients' willingness to change their medication	GE	4	y	y	y
		Patients' willingness to change their life style	UK	3	y	y	y
	Patient behaviour	Patient do not bring their medication packages for "brown bag review"	GE	3	y	y	y
		Patients do not report their medication completely	GE	5	y	y	y
		Long-term stays abroad of patients with migration background	GE	4	n	n	n
		Non-adherence of patients	PL	4	n	n	n
	Patient motivation	Patients' interest in medication-related topics	GE	2	y	y	y
		Problems to motivate patients to physical exercise	NW	4	y	n	n
		Travel distance for patients to the GP practice	NW	5	n	n	n
	Beliefs and knowledge	Patients' comprehension / memory of information given about medication	GE	4	y	y	y
		Language barriers/low literacy level	GE	4	y	y	y
			NL	5	n	n	n
		Awareness esp. among younger patients (for the need to have their medication lists with them)	GE	4	y	y	y
		Too many (contradictory) information sources for patients about medication	GE	4	y	y	n
		Patients' disease concept /knowledge about the disease	NW	4	y	n	n
			PL	4	y	y	y
			UK	1	y	y	y
	Patient needs	Patients needing home visits/living in nursing homes	GE	4	y	y	n
		Social support for patients	GE	4	y	n	n
Capacity for organisational change	Assistance for organisational changes	Identification of the best target group for SMC (those patients who really profit from the recommendation)	GE	1	y	y	y
		Availability of the target group/patients to schedule appointments for SMC	GE	4	n	n	n
		Amount of information GPs receive	NW	5	n	n	n
	Organisational culture	Level of practice organisation (e.g. existence of recall system, disease management programs, scheduled appointments)	GE	2	y	y	y
		Organisation of care for CVRM patients	NL	4	y	n	n
	Priority of necessary change	Priority of the targeted patient group in the municipality	NW	4	y	y	y
	Regulations, rules, policies	Secrecy among volunteers	NW	4	y	n	n
Incentives and resources	Financial incentives and disincentives	Reimbursement for SMC	GE	2	y	y	n
	Availability of necessary resources	Time	NL	3	y	n	n
			GE	1	y	y	n
			PL	1	y	n	n
			NW	1	y	y	y
			UK	4	y	n	n
		Workload / high number of targeted patients	NL	5	n	n	n
		Availability of resources to motivate and inform patients	UK	3	y	y	y
			PL	4	y	y	y
		Availability of well trained staff	PL	2	y	y	y
		Availability of voluntary organizations	NW	5	y	n	n
		Financial restraints in the healthcare budget	NW	4	y	n	n
			UK	4	y	n	n
		Recruitment of volunteers	NW	2	y	y	y
		Priority of the targeted patient group in specialist healthcare	NW	4	y	y	y
		Lack of prescriptive information	UK	2	y	y	y
	Information system	Functions and compatibility of the practice software	GE	1	y	y	n
			NL	3	n	n	n
		Integration of MRC scale into practice software	PL	1	n	n	n
		Availability and practicability of software for medication checks (medication reviews)	GE	4	y	y	y
		Typing errors (medication lists)	GE	4	y	n	n
		Administration (documentation is time-consuming)	NL	5	y	n	n

Professional interactions	Referral processes	Prescriptions by other physicians	GE	2	y	y	y
		Feedback from specialists	PL	5	y	n	n
		Knowledge about services/referral options in changing health care system	NW	2	y	y	y
			UK	5	y	y	y
	Team skills and processes	Involvement of health care assistants / delegation of tasks	GE	1	y	y	y
		Degree of teamwork	GE	4	y	y	y
		Presence of practice managers	NL	5	n	n	n
		Changes in staff / continuity of care	NL	5	n	n	n
			GE	2	n	n	n
			NW	5	y	n	n
		Well-being of the team members	NL	4	y	n	n
Social, political and legal factors	Contracts	Contracts between pharmacies and pharmaceutical industry (patient cannot distinguish generics)	GE	1	y	y	y
		Participation of practices in care groups	NL	4	y	n	n
	Payer and funder policies	Funds for physical exercise programs	NL	5	y	n	n
Individual health care professional factors	Skills needed to adhere	Knowledge of guidelines (medication reviews)	GE	5	y	y	n
		Recognition of adverse drug reactions	GE	5	n	n	n
		Recognition of depression in elderly patient	NW	4	y	n	n
		Communication skills	PL	4	y	n	n
		Skill to raise and discuss the issue of weight with patients in an acceptable way	UK	4	y	y	y
		Skill to assess patients' willingness to change	UK	5	y	y	y
		Use of a patient-friendly language (SMC)	GE	5	y	y	y
		Skill to effectively measure waist circumference	UK	5	y	y	y
		Experience with the targeted patient group	NL	5	n	n	n
			NW	2	n	n	n
	Attitudes and cognitions / agreement with recommendation	Attitude of GPs towards shared decision making	GE	5	n	n	n
	Attitudes and cognitions/intention and motivation	Travel distance for GPs to attend courses	NW	5	n	n	n
		Priority of referrals	NW	5	n	n	n
		Interest of GPs in the targeted patient group	NW	2	y	n	n
		Ownership to the implementation plan	NW	5	y	y	y
	Attitudes and cognitions / expected outcome	Lacking conviction that recommendations are necessary because of lacking evidence	GE	4	n	n	n
	Attitudes towards guidelines in general	Attitude of GP towards standardisation of health care/guidelines	GE	4	n	n	n
	Awareness and familiarity	Awareness for the problem on the part of the practice team	GE	3	n	n	n
	Behaviour/Nature of behaviour	Time for change/relapse to former routines	GE	2	y	y	y
	Knowledge about own practice	Habituation/attention for changes in well-known patients	NW	5	n	n	n
	Professional behaviour/Capacity to plan change	Priority of the patient group in private health care	NW	2	y	y	n
		Priority of the patient group in GP practices	NW	2	y	n	n
Determinants, which could not be assigned to any checklist category	Determinants related to the research design	Complexity of the patient questionnaire	NL	5	n	n	n
		Uncertainty about the beginning and end of the intervention	NL	5	n	n	n
		Letter is lost	NL	5	n	n	n
		Availability of GPs by phone	NW	5	n	n	n
	Other	Confusion of the term "depression care manager" with other services	NW	5	n	n	n

* T = trial, F = Frequency, IB = identified before, PB = prioritized before, IA = intended to addres ° Frequency scale: 1=strong issue in almost all interviews, 2=strong issue in a few interviews, 3=side issue in almost all interviews, 4 = side issue in a few interviews, 5 = single statement
