# Supplementary material for: Process evaluation of five tailored programs to improve the implementation of evidence-based recommendations for chronic conditions in primary care
Source: Implement Sci. 2016 Sep 13;11:123. doi: 10.1186/s13012-016-0473-8 (PMC5022166; doi:10.1186/s13012-016-0473-8)
Supplement: Supplementary file 5 — Results of the qualitative analysis (aspects related to the use of the strategies). (PDF 166 kb) [file 13012_2016_473_MOESM5_ESM.pdf]

Process evaluation of five tailored programs to improve the implementation of evidence-based recommendations for chronic conditions in primary care

C. Jäger, J. Steinhäuser, T. Freund, R. Baker, S. Agarwal, M. Godycki-Cwirko, A. Kowalczyk, E. Aakhus, I. Granlund, J. van Lieshout, J. Szecsenyi and M. Wensing

Additional file 5: Results of the qualitative analysis (aspects related to the use of the strategies)

Assignment to the TICD checklist category		Reasons why a strategy was used/not used or considered helpful / unhelpful (themes derived from inductive analysis)	Example quotations
Individual health care professional factors	Knowledge and skills	Extent to which the strategy conveys useful / new knowledge and skills	• *The workshop was helpful, (…) I could answer questions of patients more easily afterwards (GE).*<br>• *I completed the web program, but I haven't got new insights (NL).*<br>• *Yes, the e-course is excellent (...) one learns techniques and that is good (NW).*
	Awareness and familiarity	Extent to which the strategy raises awareness	• *I already did this before the intervention, but now I am more aware of it (NL).*<br>• *Most of us knew this from before, but the discussion increased my awareness (NW).*<br>• *It increased my awareness and perhaps I am more likely to ask patients to demonstrate the technique of inhalation (PL).*
	Intention and motivation	Extent to which the strategy motivates the target group	• *This helped us a lot. The staff was more motivated and as you can see we have realized most of the issues we have elaborated (GE).*<br>• *We didn't know what this was all about, but the information inspired me to take the CME-course (NW).*
		Extent to which the target group feels responsible for using the strategy	• *It certainly should be done because it is our duty (PL).*<br>• *It is not a task of the practice nurse to identify a depression (NW).*
		Extent to which the target group sees a need or priority for the strategy	• *We do all this already and this is why there was nothing to change (GE).*<br>• *I've looked at them, but I don't use them. I don't think that the content of the referral affects our partners' priorities (NW).*
	Attitude towards guidelines in general	Extent to which the strategy finds a balance between structuring care and allowing flexibility/individual care	• *I need the checklist only if I don't have knowledge. I think you should trust the doctors more! They have knowledge and they don't need checklists (GE).*<br>• *The checklist with information on what should be done is not necessary. I do not hold rigidly to what is written there (NL).*<br>• *The use of a check list probably will not improve care at all (PL).*<br>• *Yes, this template was useful. It helped me to structure my referral. (NW).*
	Professional behaviour / self-monitoring and feedback	Extent to which the strategy includes reminders / ongoing support for the requested behaviour	• *We gave them the bag and then it worked quite well. The patients even reminded us, when we had forgotten about it (GE).*<br>• *The majority of the patients really appreciated the literature, they appreciated that it wasn't just a leaflet given to them, but that there was follow-up (UK).*
		Extent to which the target group was reminded of the offered strategy / to which knowledge transfer was sustainable	• *I haven't paid attention to the websites, from now on I will do that (NL).*<br>• *I don't remember that I received this kind of information (NW).*<br>• *This was already some months ago. In the meantime, other information has been stored on my hard disk (GE).*
Professional interactions	Communication and influence and team processes	Extent to which the strategy implies interactive elements, exchange of experiences, team work or feedback	• *What was most helpful for us: the experiences of the other colleagues (GE).*<br>• *That was pretty confrontational, which acted as a wake-up call (NL).*<br>• *The meeting improved our communication with other healthcare professionals in the community (NW).*
Patient factors	Patient needs, preferences and knowledge	Extent to which the strategy is valuable for and accepted by patients	• *The patients often addressed us because of the posters you gave us. This was something the patients concentrated on (GE).*<br>• *This have been particularly useful for patients who live some distance from my practice (NW).*<br>• *It was their goal and not something that I set, it was what they defined and what they wanted to get to (UK).*

Guideline factors / recommended clinical intervention or behaviour	Accessibility	Extent to which the strategy is at hand (e.g. integrated into practice software)	• *If the checklist was digital, we would probably use it (GE).*<br>• *The referral forms were OK, but to use them, they should have been integrated in my practice software (NW).*<br>• *The inhaler devices were always on my desk, so I used it (PL).*
		Extent to which the target group for the strategy is present / defined	• *After the outreach visit we made a plan and decided that some patients could contact me or the practice staff regularly. The patients who received this service were very happy with it (NW).*<br>• *I didn't have to recommend the psychical exercise group because there were no patients with mild depressive symptoms (NL).*<br>• *Patients who do not attend consultations due to depression are rare in my practice (NW).*
	Feasibility	Extent to which the strategy is intuitive / functional/easy to use	• *I did not give patients the information card because there was an error on the card (NL).*<br>• *We used the booklets mainly because they had photographs in it (UK).*
		Extent to which the strategy requires the use of technologies (e.g. internet connection)	• *Almost no one of our patients was able to use this tablet. The health care assistant had to read it to them (GE).*<br>• *The internet connection was slow, so we stopped using the online lectures, printing the PDF hand-outs instead (NW).*<br>• *Patients in my group are not really into twitter, even if they had internet they wouldn't use twitter (NL).*
	Effort	Extent to which the use of the strategy demands time or helps to save time	• *I did not look at the web program, I was too busy (NL).*<br>• *Meeting with relatives is very useful and saves me a lot of time in my practice, because various family members know different things about the patient (NW).*
	Compatibility	Extent to which the strategy fits with pre-existing habits / structures and personal preferences	• *We are not using the posters any more, because we generally don't like it, if there is so much paper on the walls in the practice (GE).*<br>• *We personally don't work with strive targets for patients. So I did not fill it out on the information card (NL).*<br>• *I don't give this kind of information, I have little experience with such services (NW).*
	Quality of evidence	Extent to which the strategy includes information about evidence	• *Very useful; when patients are informed that my recommendations are consistent with national guidelines, they are more receptive (NW).*<br>• *Yeah, I would (use it), because it is a resource that's designed around the NICE guidelines (UK).*
Incentives and resources	Availability of necessary resources	Extent to which the strategy provides necessary resources / useful tools	• *The internet tools you showed us on the tablet, to check side effects quickly, that was helpful (GE).*<br>• *I've read the folder together with the patient, this project has really provided valuable tools for the GP (NW).*
	Financial incentives and disincentives	Extent to which the strategy includes reimbursement / requires personal commitment	• *It is a strong motivation that the activities merit for my speciality (NW).*<br>• *I should do it at home and that does not motivate me at all (NL).*<br>• *It (the workshop) took too much time. I had to close my practice and reschedule all appointments (GE).*

GE = Germany, NL = The Netherlands, NW = Norway, UK = United Kingdom, PL = Poland
