# Supplementary material for: Process evaluation of five tailored programs to improve the implementation of evidence-based recommendations for chronic conditions in primary care
Source: Implement Sci. 2016 Sep 13;11:123. doi: 10.1186/s13012-016-0473-8 (PMC5022166; doi:10.1186/s13012-016-0473-8)
Supplement: Supplementary file 6 — Results of the qualitative analysis (other strategies not used in the implementation programs). (PDF 209 kb) [file 13012_2016_473_MOESM6_ESM.pdf]

Process evaluation of five tailored programs to improve the implementation of evidence-based recommendations for chronic conditions in primary care

C. Jäger, J. Steinhäuser, T. Freund, R. Baker, S. Agarwal, M. Godycki-Cwirko, A. Kowalczyk, E. Aakhus, I. Granlund, J. van Lieshout, J. Szecsenyi and M. Wensing

Additional file 6: Results of the qualitative analysis (other strategies not used in the implementation programs)

Classification of strategies		Strategy (original phrasing)	C	IB*	PB**
Induce organizational change on practice / institutional level	Integration of processes in existing structures	Combine structured medication counseling with other "recall-appointments"	GE	y	y
	Revision of roles / tasks of staff	Delegation of tasks to nurses	PL	y	y
		Assign one person to the task	NW	y	y
	Facilitate access to care	Hotline for selected patients	NW	n	n
	Change records systems	Integrate resources into the practice software	GE	n	n
			NW	y	n
		Provision of software for medication checks, integrated in practice software	GE	y	n
		Contact software providers to change templates (medication lists)	GE	y	n
		Tablet PC with connection to practice software for home visits	GE	n	n
	Other	Provide a checklist focusing more on re-organization of care on individual level	PL	n	n
Change on system or regional level	Improve health technologies	Insurance card with stored medication list	GE	y	n
	Change tasks and responsibilities of HCP	Specialist health care should offer group psychotherapy for elderly patients	NW	n	n
		Specialists in private practice could be requested a certain proportion of elderly on their patient list	NW	n	n
	Support of referral processes	Improve communication processes at health care interfaces	NW	n	n
		Facilitate contact with pulmologists	PL	y	n
	Various	Target other locations (e.g. mosques, temples, ante-natal clinics, places where elderly people meet)	UK	n	n
			NW	y	y
		More time for patient consultations	PL	y	n
		Standardisation of medication lists on district level	GE	n	n
		Change of laws: no trade names on medication packages	GE	y	n
Ongoing support	Reminders	Reminders about the project (e.g. by email, telephone or practice visits) during intervention period	GE	n	n
			NL	n	n
			NW	n	n
	Repeated training	Instruct patients repeatedly how to use inhalers	PL	y	n
		Repeated training in motivational interviewing	NL	y	Y
rsonal educational interventions	External feedback (audit and feedback)	Send the results of this research to the practice nurses	NL	n	n
	Induce knowledge exchange between peers	Supervision of GPs by psychiatrists in collaborative care	NW	y	n
		Medication reviews / feedback by pharmacists	GE	y	n
		Case conferences (in quality circles or via video)	GE	y	n
		Involve local psychiatrists into outreach visits to increase enthusiasm	NW	n	n
		Practice nurses should give each other mutually feedback on motivational interviewing	NL	n	n
	Assess existing knowledge	Ask patients to actively report their knowledge about their medication	GE	n	n
	"Training" without further specifications	Training courses for patients on COPD	PL	y	y
		Course in communication for people who volunteer to support elderly patients with depression	NW	y	n
		Training courses for physicians and nurses	PL	y	y
		Training for newly employed GPs (in order to maintain knowledge in the municipality)	NW	n	n
		Train more staff, e.g. junior staff	UK	y	n
		Single-day course for GPs (as alternative to online-course)	NW	n	n

Provision of educational materials	Provision of materials/tools	Patient folders with relevant documents	GE	y	n
			NW	y	n
		Leaflets (instead of posters)	GE	y	y
			PL	y	y
		Posters (in addition to leaflets)	NW	n	n
		Paper-based information instead of infotool on tablet PC	GE	y	y
		Provision of spirometers	PL	y	n
		Provide information material for patients on COPD	PL	n	n
		List of healthy snacks	UK	y	n
		Integrate information on web-sites that GPs already use	NW	y	n
	Provision of evidence	Provide information on statistics of COPD	PL	N	n
		Provide information on clinical usefulness of the interventions and recommendations	NW	Y	Y
Improve cost-benefit-ratio	Increase benefits	Provide financial incentives	PL	y	n
			NW	n	n
		Highlight the benefits for the GPs / more intensive marketing	NW	y	y
	Reduce costs	Simplify / shorten the intervention	NL	n	n
Adapt strategies on individual patient level	Lingual adaptions	Translation (of booklets)	UK	y	n
	Cultural adaptions	Food-recommendations adapted to cultures	UK	y	n
	Personal adaptions	Personalize life-style advice	NL	y	y
Improve development and delivery of the intervention	Involvement of the target group in the development	Involve the target group into intervention development	NW	n	n
			NL	n	n
		Acquire commitment from municipalities and GPs	NW	n	n
	Improve information about the intervention	Better guidance / guideline for the intervention / more examples	NL	n	n
			NW	y	n
		Have one fix contact person from the research team	NL	n	n
		Inform HCP about the content of the patient questionnaire for data collection	NL	n	n
	Other	Expand intervention period	NL	n	n

* C = country, ** IB = Identified before, *** PB = prioritized before; y = yes, n = no
